# REGENERATIVE MEDICINE – INITIATIVE À LA CARTE A remedy to the blood thirsty count Dracula?

**DOI:** 10.1111/j.1582-4934.2008.00392.x

**Published:** 2008-06-28

**Authors:** Yrjö T Konttinen, Gerjo van Osch

**Affiliations:** Dept Medicine, Institute of Clinical Medicine Helsinki University Central HospitalBiomedicum Helsinki P.O.B. 700, FIN-00029 HUS Finland*e-mail:*Yrjo.konttinen@helsinki.fi; Dept Orthopaedics & Otorhinolaryngology Erasmus MC, University Medical Center Rotterdam Dr Molewaterplein 503015 GE Rotterdam The Netherlands*e-mail:*g.vanosch@erasmusmc.nl


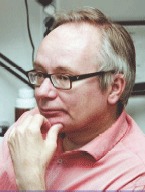



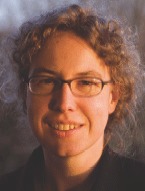


Count Dracula living in the beautiful Carpathian Mountains in Transylvania, splendidly played by the late Béla Lugosi, was maintaining himself with repeated blood meals. Due to the limited lifetime of red blood cells he needed this form of substitution therapy over and over again. It was not known then that transplantation of hematopoietic stem cells would have provided a permanent cure. To protect European Union citizens from such bad, not to say deadly, table habits, the Strasbourg-based European Science Foundation has recently activated a program solely dedicated to Regenerative Medicine, REMEDIC.

Early attempts to regenerate tissue were done by the former generation scientist Victor Frankenstein and results have been widely publicized. He used extensive transplantation and implantation, but as the rules of modern regenerative medicine were not followed and the powers of the stem cells had not been revealed yet, the outcome, ‘the Creature Frankenstein’, fell far from a perfect creature although many of us find him quite sympathetic—at least on the white screen.

Let us check how Regenerative Medicine is seen in EU as that provides some idea about the impetus and impact of this field of study in European science. Indeed, the 7^th^ EU Framework program will probably soon announce not less than three large-scale integrating actions in this field:

1. Cell therapy for tissue and organs.
2. Regeneration of tissue using biocompatible materials and cells.
3. Activation of endogenous cells as an approach to regenerative medicine.

This is quite remarkable as each of these actions will probably be funded with €12 million. REMEDIC forms an excellent platform for such an application as the scientists participating in the action are involved in several clinical trials aiming to clinical transfer of already established findings and there is also a considerable input from former FP7 calls into this action. The Steering Board is open for participation from research-active basic and clinical scientists and commercial companies in this field, also from non-committed countries.

What does ‘non-committed’ mean in this context? European Science Foundation run Research Networking Programs work according to à la carte principle. All EU membership and associated countries get ‘the menu’ and either they pick up their choice – or they do not. If they make a selection, they naturally are ready to pay for it, too. As the ‘chef’ was a Finn, much to our regret, Italy decided not to participate, hopefully not due to the comments of the Italian prime minister Silvio Berlusconi on the Nordic food. REMEDIC was selected by 13 different member countries, which participate by funding:

- Fonds zur Förderung der wissenschaftlichen Forschung in Österreich (FWF)
  Austrian Science Fund, **Austria**
- Fonds voor Wetenschappelijk Onderzoek—Vlaanderen (FWO), **Belgium**
- Forsknings- og Innovationsstyrelsen—Forskningsrådet for Sundhet og Sygdom (FSS) Forsknings- og Innovations styrelsen, **Denmark**
- Suomen Akatemia, **Finland**
- Deutsche Forschungsgemeinschaft (DFG), **Germany**
- Nederlandse Organisatie voor Wetenschappelijk Onderzoek (NWO), **The Netherlands**
- Organisation for Health Research and Development (ZonMw) **The Netherlands**
- Norges Forskningsråd, **Norway**
- Fundação para a Ciências e a Tecnologia (FCT) Headquarters, **Portugal**
- National University Research Council (NURC), **Romania**
- Slovenská Akadémia Vied (SAV), **Slovakia**
- Consejo Superior de Investigaciones Cientificas (CSIC), **Spain**
- Ministerio de Educacion y Ciencia (MEC), **Spain**
- Vetenskapsrådet (VR)— Swedish Research Council, **Sweden**
- Swiss National Science Foundation (SNSF), **Switzerland**

Unfortunately this à la carte principle also means that

1. each participating country is entitled to only one official partner
2. non-committed countries can not be directly funded by REMEDIC

But as always, it is not as bad as it sounds. Non-commitment does not exclude you from participation in some of the activities of the REMEDIC. In particular, we welcome active participants and partners into this action, in particular to our joint application to the 7^th^ FP on the use of mesenchymal stromal (stem) cells in the biology, pathology and treatment of heart failure after myocardial infarction. Other joint applications will follow, focusing on other applications, including orthopaedic and gastrointestinal.

REMEDIC has already organized a workshop on ‘Regenerative Medicine’ in April in Madrid as part of the European Orthopaedic Research Society and another one in Strasbourg in France. Work includes mapping of needs and resources, a register of companies in file for knowledge transfer and clinical translation, state-of-the-art reviews and chapters planned also for this forum, guide to the rules and regulations like good laboratory praxis, good manufacturing praxis and quality controls too often even today neglected by serious scientists in their experimentation, workshops on regenerative medicine and naturally short-term scientific visits and exchange visits. In particular we try to promote knowledge and understanding of the potential and limitations of regenerative medicine ‘by doing’. This will happen in form of joint projects and networking with scientists, colleagues and future friends.

Regenerative medicine is engaged with stem cells, which have an almost unlimited proliferation potential accompanied by an ability to differentiate. Thus, hematopoietic and mesenchymal stem (or stromal) cells (MSC) form an essential element in regenerative (or reparative) medicine, including guided regeneration. Instead of implantation of a prosthetic device or transplantation of donor organ, regenerative medicine relies on the versatile genetic information stored tightly packed in the cell nuclei, from where it is released (**=** copied and then translated into proteins) to regulate the repair of cells, tissues and organs in an intelligent way.

When such multipotent stem cells are harvested from autologous sources, immunological rejection and the burden of the use of immunosuppressive (cytotoxic) drugs are avoided. Endogenous stem cells can be activated with proper growth and differentiation stimuli to maintain or augment bodily functions. Subcutaneous fat tissue forms an autologous (personal) MSC source, which can be simply collected using a needle for fat aspiration. Cells isolated are allowed to expand and then driven to differentiate as such, in tissue engineering devices or bioreactors before applying back to body as cells, tissues or organs, either locally or systemically into circulation. They help the target site(s) to regenerate so that structure and function are not replaced but restored.

Restriction to the use of hematopoietic and mesenchymal stem cells avoids the ethical issues associated with the use of embryonic stem cells. Autologous stem cells have a known source of origin and donor cells cannot transmit any new infections or prions to the recipient. Such cells can be used at once or stored in biobanks for eventual future use. Versatility of the cell-based therapies can be enhanced by genetic manipulation of the cells in the laboratory, coding of a missing protein such as hormone or enzyme for the life-time of the recipient. For experimental work markers useful in tracing of the transferred cells can be added to study the long-term effects.

Regenerative medicine provides new insight in realms comprising cellular proliferation, effects of humoural and matrix signalling on cells, angiogenesis, tissue remodelling, naïve and adaptive immunity and other basics in cell biology. Still, regenerative medicine is in its infancy and to facilitate the process, 13 EU countries have joined forces in an attempt to improve networking and clarify where the frontiers and future needs are in this complex multidisciplinary high-tech, high-medicine field. In general, in regenerative medicine, apart from input in basic science, proof of principle experiments and randomized clinical trials are ahead, for which separate support will be sought for. In the most futuristic views, the human body is seen to undergo repeated and adequately timed maintenance of the failing body parts with cycles of healing cell therapies.

REMEDIC will be running from April 2008 to April 2013. Chair can be contacted at yrjo.konttinen@helsinki.fi and more information on the members and activities is already available on the web of the European Science Foundation; program home pages are being reconstructed.

